# Sensitive Electrochemical Detection of Carcinoembryonic Antigen Based on Biofunctionalized Nanochannel Modified Carbonaceous Electrode

**DOI:** 10.3390/molecules29040858

**Published:** 2024-02-15

**Authors:** Yucheng Zhou, Hongxin Wang, Fengna Xi, Chao Lu

**Affiliations:** 1 General Surgery, Cancer Center, Department of Gastrointestinal and Pancreatic Surgery, Zhejiang Provincial People’s Hospital, Hangzhou Medical College, Hangzhou 310014, China; drzhouyc@163.com; 2 Key Laboratory of Gastroenterology of Zhejiang Province, Hangzhou 310014, China; 3 School of Chemistry and Chemical Engineering, Zhejiang Sci-Tech University, Hangzhou 310018, China; 202230107404@mails.zstu.edu.cn

**Keywords:** electrochemical detection, immunosensor, vertically aligned mesoporous silica film, carcinoembryonic antigen, high sensitivity

## Abstract

The convenient construction of carbon-based electrochemical immunosensors with high performance is highly desirable for the efficient detection of tumor biomarkers. In this work, an electrochemical immunosensor was fabricated by integrating a biofunctionalized mesoporous silica nanochannel film with a carbon-based electrode, which can enable the sensitive determination of carcinoembryonic antigen (CEA) in serum. The commonly used carbonaceous electrode, glassy carbon electrode (GCE), was employed as the supporting electrode and was pre-treated through electrochemical polarization to achieve the stable binding of a vertically ordered mesoporous silica film with amino groups (NH_2_-VMSF) without the use of any adhesive layer. To fabricate the immunorecognition interface, antibodies were covalently immobilized after the amino groups on the outer surface of NH_2_-VMSF was derivatized to aldehyde groups. The presence of amino sites within the high-density nanochannels of NH_2_-VMSF can facilitate the migration of negatively charged redox probes (Fe(CN)_6_^3-/4-^) to the supporting electrode through electrostatic adsorption, leading to the generation of electrochemical signals. In the presence of CEA, the formation of immunocomplexes on the recognitive interface can reduce the electrochemical signal of Fe(CN)_6_^3-/4-^ on the supporting electrode. Based on this principle, the sensitive electrochemical detection of CEA was achieved. CEA can be determined to range from 0.01 ng mL^−1^ to 100 ng mL^−1^ with a limit of detection of 6.3 pg mL^−1^. The fabricated immunosensor exhibited high selectivity, and the detection of CEA in fetal bovine serum was achieved.

## 1. Introduction

Tumors are malignant diseases that pose a serious threat to human health. Tumor cells have the ability to proliferate indefinitely and infiltrate, which can disrupt normal tissue structure and function and lead to organ failure or even death [1]. The early detection of tumors and their timely treatment can effectively improve the cure rate and survival rate. Tumor biomarkers play an important role in the early screening, diagnosis, and treatment of tumors [2,3,4]. Specifically, tumor biomarkers are specific molecules or substances whose levels in the body of tumor patients are usually related to the presence, development, and treatment response of tumors. Thus, the efficient detection of tumor biomarkers has significant clinical significance and can help in the early diagnosis of tumors, monitoring treatment effectiveness, and assessing the risk of recurrence, providing strong support for the treatment of tumor patients. For example, carcinoembryonic antigen (CEA) is a commonly used tumor biomarker, with a typical range in human serum being less than 5 ng/mL [5,6,7,8]. Under normal circumstances, CEA is produced during fetal development and is only present in very small amounts in the adult body. However, certain types of tumors, such as colorectal cancer, breast cancer, and lung cancer, can cause the excessive production of CEA, leading to elevated levels in the blood. Therefore, the fast and sensitive determination of the level of CEA in the serum is of great significance.

Immunoassays are one of the most common methods for CEA detection [9,10,11]. This method involves the use of specific antibodies to bind with CEA, forming an immunocomplex, which is then quantitatively analyzed using methods such as fluorescence, enzyme labeling, radioisotopes, electrochemistry, and electrochemiluminescence [12]. Compared to others, electrochemical immunoassays have advantages such as simple instrumentation, rapid detection, convenient operation, and high selectivity [13,14,15]. Various carbon-based electrodes are widely used as basic electrodes in electrochemical biosensors due to their high chemical stability, low cost, wide potential window, and good biocompatibility [16,17,18,19]. The convenient construction of carbon-based immunosensors with high performance is highly desirable.

In recent years, porous materials have attracted extensive research interest due to their large surface area, demonstrating great applications in adsorption, separation, catalysis, sensors, and other fields [20,21,22,23]. Unlike bulk porous materials, porous film has a higher compatibility with two-dimensional (2D) planar electrodes. For instance, single conical nanopores on polyethylene terephthalate (PET) films can be employed as nanofluidic diodes with highly nonlinear current–voltage characteristics, offering a unique possibility to construct different biosensors [24,25]. Furthermore, vertically ordered mesoporous silica film (VMSF) has received considerable attention [26,27,28,29]. VMSF has high-porosity, vertically ordered nanochannels with a size from 2 to 3 nm [30,31,32,33]. These characteristics give VMSF a fast mass transfer ability and selectivity based on size and charge [32,33,34,35]. For instance, the ultra-small nanochannel of VMSF can exclude larger proteins or particles, exhibiting size sieving effects [36,37,38]. A signal-decreased sensing mode can be fabricated by generating large-sized substances during the detection process, hindering the migration of small molecular probes into the nanochannels [39,40,41]. Due to the unique three-dimensional (3D) structure of VMSF, its functionalization can be considered from three aspects including the modification of the nanochannels, outer surface, and the supporting electrode. For example, VMSF with functional groups can be synthesized in one step by using siloxane containing functional groups (e.g., amino or thiol groups) [30]. Furthermore, VMSF can be bio-functionalized on the external surface of VMSF to introduce recognition ligands [42,43]. At the same time, the modification of the supporting electrode to improve its electrochemical activity also contributes to the improvement of the sensing system. Thus, the VMSF-modified electrode exhibits excellent anti-fouling, charge-selective permeability, and independent functional regions, demonstrating great potential for constructing a biosensing platform and the direct electrochemical analysis of complex samples.

In this work, an electrochemical immunosensor was fabricated by integrating silica nanochannel film with a highly active carbon-based electrode, which can achieve the sensitive determination of CEA in serum. The supporting glassy carbon electrode (GCE) was pre-treated by electrochemical polarization (p-GCE). The vertically aligned mesoporous silica film with amino groups (NH_2_-VMSF) was directly integrated onto the surface of p-GCE using the electrochemically assisted self-assembly (EASA) method without the use of another adhesive layer. The derivatization of amino groups on the outer surface of VMSF enabled the covalent immobilization of recognition antibodies, followed by further blocking with bovine serum albumin (BSA) to block the non-specific sites, establishing the recognitive interface. During CEA detection, the formation of immunocomplexes reduced the electrochemical signal of redox probes on the supporting electrode, leading to the highly sensitive electrochemical detection of CEA. The VMSF-based immunosensor has the advantages of ease of preparation, high sensitivity, and good selectivity.

## 2. Results and Discussion

### 2.1. Strategy for Fabrication of Immunosensor by Growing VMSF on p-GCE 

**Figure 1:**
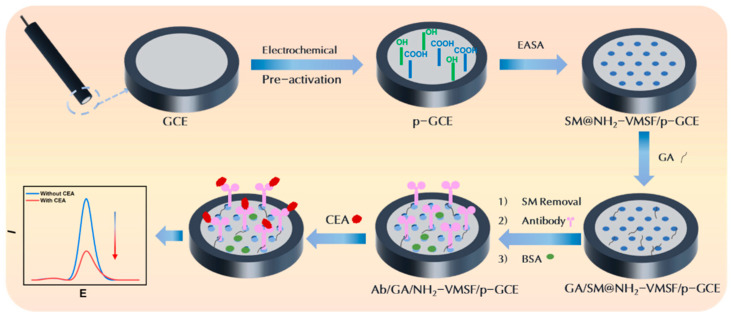
Schematic illustration of the construction of the immunosensor and the electrochemical detection of CEA.

As shown in Figure 1, GCE was used as the supporting electrode and further integrated NH_2_-VMSF after electrochemical polarization treatment. As one of the electrodes extensively used in electrochemical sensors, GCE possesses high chemical stability, a broad potential window, and excellent biocompatibility. However, VMSF cannot directly form a stable bond with GCE. As illustrated in Figure 1, p-GCE was obtained through the simple and environmentally friendly electrochemical polarization. Typically, GCE undergoes anodic oxidation at a high potential (+1.8 V), which results in the etching of the GCE surface, generating abundant defects and oxygen-containing functional groups. Subsequently, cathodic polarization at low potential (−1.3 V to +1.25 V) partially reduces these oxygen-containing groups, restoring the sp^2^ structure on the electrode surface and thereby enhancing the electrode’s conductivity.

Then, VMSF was directly grown on p-GCE using the EASA method. When a negative voltage or current is applied to the electrode, the electrochemical reduction of H^+^ and H_2_O leads to an increase in the pH at the electrode–solution interface. This in situ pH increase induces the self-assembly polymerization of siloxanes, consequently resulting in the formation of surfactant-templated silica nanochannels on the electrode surface [44]. This approach can achieve the fast growth of VMSF. Simultaneously, the -OH groups on the p-GCE surface could form Si-O covalent bonds with VMSF through a co-condensation reaction with Si-OH groups, thereby enhancing the stable adhesion of VMSF. As an amino-functionalized siloxane precursor was used, the resulting VMSF was rich in amino functional groups and labeled as NH_2_-VMSF. After the completion of nanochannel growth (SM@NH_2_-VMSF/p-GCE), the SM-filled nanochannels can be simply removed by immersing the electrode in a hydrochloric acid–ethanol solution, resulting in an open nano-channel array (NH_2_-VMSF/p-GCE).

To construct the immunosensing interface, antibody (Ab) was immobilized on the external surface of the VMSF. The amines were firstly modified using glutaraldehyde (GA), successfully introducing aldehyde groups, which allowed for the covalent immobilization of the Ab on the VMSF. Specifically, GA derivatization was performed on SM@NH_2_-VMSF/p-GCE without removing the SMs, preventing GA from entering the channels and affecting their permeability. Subsequently, SMs were removed and GA/NH_2_-VMSF/p-GCE was obtained. The antibody (Mw 150 kDa) was then covalently immobilized through the reaction between the aldehyde groups and the amine groups in the antibodies (Ab/GA/NH_2_-VMSF/p-GCE). After blocking the non-specific sites with BSA, the immunosensor (BSA/Ab/GA/NH_2_-VMSF/p-GCE) was obtained. In comparison to commonly used methods (e.g., biotin- and avidin-based directional immobilization), the construction of the immunosensor offers the advantages of simplicity, fast fabrication, and cost effectiveness. 

The constructed immunosensor enables the electrochemical detection of CEA using the redox probe Fe(CN)₆^3-/4-^ in the solution. Due to the high-density nanochannels in NH_2_-VMSF, the Fe(CN)_6_^3-/4-^ probe in the solution can efficiently migrate to the underlying electrode, generating an electrochemical signal. Upon binding to CEA (molecular weight, Mw, 180 kDa), the resulting antibody–antigen complex might hinder the migration of Fe(CN)_6_^3-/4-^ to the underlying electrode surface and increase the interfacial resistance, leading to a decrease in the electrochemical signal. Based on this principle, the electrochemical detection of CEA can be achieved.

### 2.2. Characterization of p-GCE and NH_2_-VMSF/p-GCE

**Figure 2:**
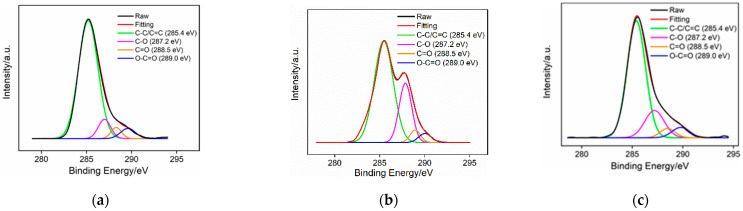
High-resolution C1s XPS spectra obtained on (**a**) bare GCE, (**b**) electrode obtained after anodic polarization, and (**c**) p-GCE.

The functional groups on the surface of the GCE before and after pre-activation were characterized using X-ray photoelectron spectrum (XPS). Figure 2 presents the high-resolution C1s spectra obtained on the GCE, the electrode obtained after anodic polarization, and the p-GCE. Four types of carbon bonds are observed including C-C/C=C (285.4 eV), C-O (287.2 eV), C=O (288.5 eV), and O-C=O (289.0 eV), corresponding to graphite (sp^2^), phenol/ alcohol/ ether, carbonyl, and carboxyl carbon atoms, respectively [45]. The contents of C-C/C=C are 81.80%, 66.10%, and 71.70% for the GCE, electrode obtained after anodic polarization, and p-GCE, respectively. These values indicate remarkable changes in the functional groups pre-activation. Specifically, the content of sp^2^ carbon significantly decreased owing to the oxidation in anodic oxidation, while the following cathodic reduction partially recovered the graphite structure. The ratios of the C-O, C=O, and O-C=O peak area relative to the C-C/C=C peak area in the above three electrodes were also calculated and displayed in Table S1 (Supplementary Materials). As observed, the content of the C-O and C=O bonds on the electrodes’ surfaces also significantly increased resulting from the oxidation during anodic oxidation. Compared to the GCE, the p-GCE exhibits a significant increase in the content of the three oxygen-containing groups relative to the graphite carbon, demonstrating a notable enhancement in the oxygen-containing groups on the surface of the p-GCE after electrochemical polarization. 

**Figure 3:**
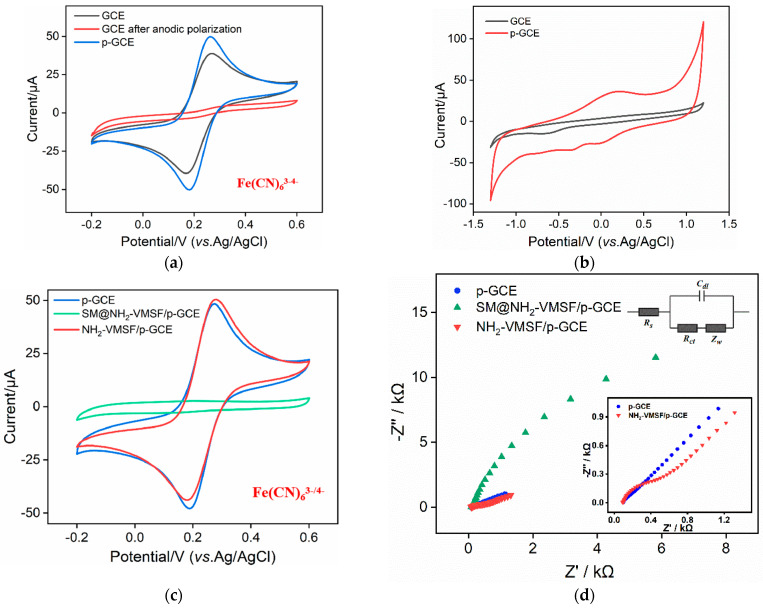
(**a**) CV curves obtained on bare GCE, the electrode after anodic polarization, and p-GCE in 0.1 M KCl containing 2.5 mM Fe(CN)_6_^3-/4-^. The scanning rate is 50 mv/s. (**b**) CV curves on GCE and p-GCE in PBS (0.1 M, pH 5.0). The scanning rate is 100 mv/s. CV curves (**c**) and EIS plots (**d**) obtained on different electrodes in 0.1 M KCl containing 2.5 mM Fe(CN)_6_^3-/4-^. The insets in Figure 3d are the equivalent circuit diagram (**upper**) and the magnified EIS plots (**below**).

To further investigate the impact of the electrochemical polarization process on the surface chemistry of the GCE, the cyclic voltammetry (CV) curves of the Fe(CN)_6_^3-/4-^ probe obtained on different electrodes were investigated. As shown in Figure 3a, distinct redox peaks were observed on the GCE. However, almost no redox peaks of Fe(CN)_6_^3-/4-^ were obtained on the GCE subjected to anodic oxidation. This suggests that the strong oxidative process at a high voltage during anodic oxidation completely inhibits the interfacial electron transfer of the probe’s molecules. After further cathodic reduction, the redox peaks of Fe(CN)_6_^3-/4-^ reappeared on the p-GCE. Additionally, compared to that on the GCE, the peak-to-peak separation of Fe(CN)_6_^3-/4-^ on the p-GCE reduced, indicating the faster charge transfer kinetics of Fe(CN)_6_^3-/4-^ on the p-GCE, demonstrating the enhanced electron transfer of the p-GCE [46,47]. 

Figure 3b displays the CV curves obtained for the GCE and p-GCE in a PBS (0.1M, pH 5.0) solution. Compared to the GCE, the p-GCE exhibited pronounced redox peaks near 0 V, attributed to the introduction of redox moieties on the electrode surface during electrochemical polarization [48,49]. Additionally, the p-GCE demonstrated significantly an increased charging current and lower anodic and cathodic hydrogen evolution potentials compared to the GCE. The effective electrode area was determined using the CV curves of Fe(CN)_6_^3-^ (Figure S1 in Supplementary Materials) based on the Randles–Sevcik equation [50]. In comparison with the bare GCE (0.029 cm^2^), the effective electrode surface area of the p-GCE increased (0.055 cm^2^). 

The electrochemical behavior of the Fe(CN)_6_^3-/4-^ probe on the different electrodes obtained during VMSF modification was studied using CV (Figure 3c) and EIS (Figure 3d). There is almost no Faraday current of the Fe(CN)_6_^3-/4-^ probe on the SM@NH_2_-VMSF/p-GCE electrode (Figure 3c). This is attributed to SMs blocking the nanochannels, thus hindering the migration of Fe(CN)_6_^3-/4^ from the solution to the underlying electrode. This phenomenon also confirms the integrity of the grown VMSF without any cracks. When the SMs are removed, the significant oxidation and reduction peaks of the probe are observed on the NH_2_-VMSF/p-GCE electrode, indicating the opening of the nanochannel. Furthermore, the peak current of Fe(CN)_6_^3-/4-^ on NH_2_-VMSF/p-GCE was higher than that on the p-GCE. As is known, VMSF contains abundant silanol groups (p*K*_a_~2), which ionize to form a negatively charged surface. The introduction of amino groups introduces positively charged sites, facilitating the migration of the negatively charged Fe(CN)_6_^3-/4-^ toward the supporting electrode, indicating charge-selective permeability. 

Consistent results were obtained from the electrochemical impedance spectroscopy (EIS) of different electrodes (Figure 3d). The insets provide a schematic illustration of the equivalent circuit (upper inset) and the corresponding enlarged EIS plots (below inset). The equivalent circuit comprises the solution resistance (*R*_s_), the Warburg impedance (*Z*_w_), the double-layer capacitance (*C*_dl_), and the apparent charge transfer resistance (*R*_ct_). As shown, the SM@NH_2_-VMSF exhibited a significantly high electron transfer resistance (*R*_et_, 35138 Ω) because of the hindered electronic transfer between the probe and the electrode when the SMs sealed the nanochannels. After removing the SMs, the *R*et of the NH_2_-VMSF/p-GCE (622 Ω) was higher than that of the p-GCE (218 Ω), resulting from the insulating silica structure.

### 2.3. Morphology Characterization of NH_2_-VMSF

**Figure 4:**
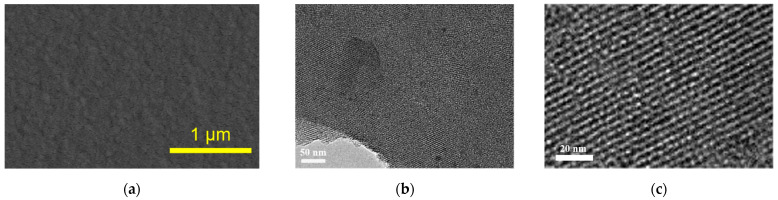
(**a**) Top-view SEM image of NH2-VMSF/p-GCE. Top-view (**b**) and cross-sectional (**c**) TEM images of NH_2_-VMSF.

The morphology and structure of the NH_2_-VMSF were characterized using scanning electron microscopy (SEM) and transmission electron microscopy (TEM), and the results are shown in Figure 4. The top-view SEM image of the NH_2_-VMSF/p-GCE displays a smooth structure with no observable cracks or defects within the observed range. From the top-view TEM image (Figure 4b), it is evident that the film exhibits uniformity and a lack of defects over a large area. As measured with ImageJ software (version 1.8.0) [44], the pore/nanochannel exhibited an average diameter of 2.7 nm (calculated using 99 pores/nanochannels) and a porosity (a fraction of the area of pores over the total area) of 42%. Figure 4c displayed the cross-sectional TEM image of the NH_2_-VMSF, revealing its long-range ordered channel structure.

### 2.4. The Feasibility for the Construction of Immunosensor

**Figure 5:**
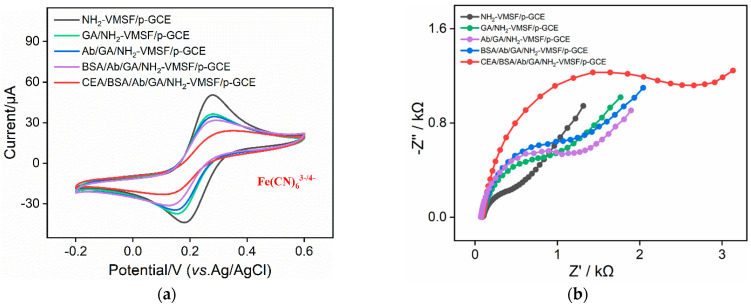
CV (**a**) and EIS curves (**b**) on different electrodes obtained in the fabrication of the immunosensor in 0.1 M KCl containing 2.5 mM Fe(CN)_6_^3-/4-^.

Changes in the electrode surface during the construction of the immunosensor were investigated using CV and EIS. Figure 5a displayed the CV curves of the different electrodes in the electrochemical probe solution. The peak current of the redox probe on the GA/NH_2_-VMSF/p-GCE slightly decreased resulting from the partial cross-linking of the amino groups by GA at the entrance of the nanochannels. The CV curve of Fe(CN)_6_^3-/4-^ on the electrode prepared by SM removal before GA derivatization was also measured (Figure S2 in Supplementary Materials). It was revealed that the electrode prepared by SM removal followed by GA derivatization exhibited a reduction in the peak current. This was attributed to the cross-linking of GA with amino groups inside the nanochannels, which affected the migration of the Fe(CN)_6_^3-/4-^ probe to the supporting electrode. When the Ab was covalently immobilized on the aldehyde surface and the BSA subsequently blocked the non-specific binding sites, the peak current of the redox probe further reduced. This is because proteins, as non-conductive substances, increase the interfacial resistance on the electrode surface. After the incubation of the immunosensor with CEA, the formation of antigen–antibody immunocomplexes decreased the electrochemical signal of the probe on the supporting electrode. Similar conclusions were verified by the EIS results, as shown in Figure 5b. The *R*et of the GA/NH_2_-VMSF/p-GCE slightly increased (1248 Ω) due to GA cross-linking with surface amino groups. The immobilization of the Ab (1522 Ω) and the subsequent BSA blocking (1672 Ω) both led to an increase in the *R*et, owing to the non-conductivity of the proteins. When binding with CEA, a higher *R*et (2920 Ω) was observed because of the formation of an immunocomplex at the recognition interface. These results confirmed the effective fabrication of the immunosensor and the decreased signal caused by immunorecognition.

### 2.5. Optimization of Immunosensor Construction Conditions

**Figure 6:**
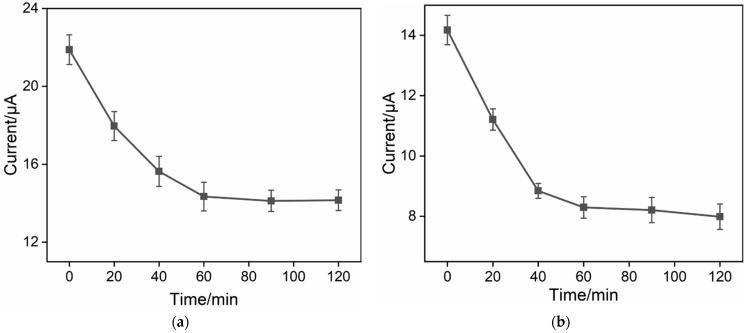
(**a**) Effect of incubation time between Ab on the aldehyde surface on the current response of the proposed immunosensor. (**b**) Effect of incubation time between the immuno-recognitive surface and the detected CEA on the current response of the proposed immunosensor. The error bars represent the relative standard deviation (RSD) of three measurements.

To further enhance the performance of the immunosensor, the duration for antibody immobilization during the immunoelectrode preparation was optimized. The immunosenors were fabricated using various antibody incubation times with the aldehyde-modified surface followed with BSA blocking. The resulting DPV peak currents for each electrode after binding with CEA are shown in Figure 6a. It can be observed that an increase in the reaction time led to a decrease in the peak current, indicating an increase in the amount of immobilized antibodies and subsequently bound CEA. When the antibody immobilization time was set at 60 min, the electrochemical signal of the electrode became stable, demonstrating that antibody immobilization on the aldehyde surface reached saturation. Thus, a 60 min antibody immobilization time was employed for further research. Additionally, the effect of the CEA binding time on the immunorecognition interface was also investigated. When the antigen incubation time was set at 60 min, the electrochemical signal reached a plateau, confirming that the antigen achieved saturated binding (Figure 6b). As a result, a 1 h incubation time between CEA and the immunosensor was used for the subsequent experiments.

### 2.6. Electrochemical Detection of CEA

**Figure 7:**
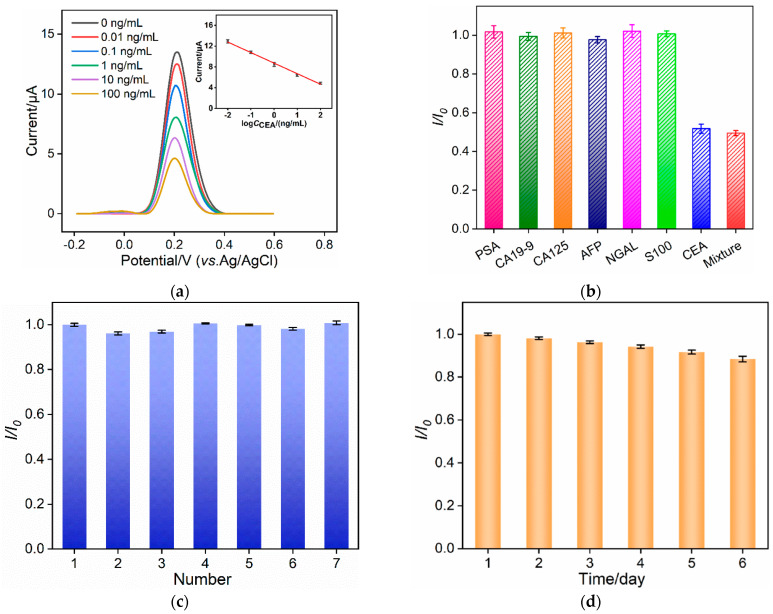
(**a**) DPV curves obtained from the immunosensors in presence of various concentrations of CEA. Inset is the corresponding calibration curve for the detection of CEA. The error bars represent the RSD of three measurements. (**b**) Relative ratio of DPV peak current before (*I*_0_) and after (*I*) incubation with PSA (10 ng/mL), CA19-9 (100 U/mL), CA125 (1 µU/mL), AFP (10 ng/mL), NGAL (10 ng/mL), S100 (10 ng/mL), CEA (1 ng/mL), or their mixture. (**c**) Relative ratio of DPV peak current obtained from seven parallel electrodes. *I*_0_ and *I* are peak current obtained on the 1st or other electrodes. The error bars represent the standard deviation of three measurements from one immunosensor. (**d**) Relative ratio of DPV peak current obtained on the immunosensor when incubated with CEA and stored at 4 °C for different days. *I*_0_ and *I* are peak current obtained before and after storage. The error bars are the standard deviation of three measurements from one immunosensor.

Under the optimal detection conditions, the performance of the constructed immunosensor for the CEA detection was investigated. Figure 7a shows the DPV signals of the electrode after incubation with different concentrations of CEA. It can be observed that with an increase in the CEA concentration, the DPV signal decreases due to an increase in the immunocomplexes on the electrode surface. As shown in the inset in Figure 7a, within the range of 0.01 ng mL^−1^ to 100 ng mL^−1^, the DPV signal (*I*) exhibits a good linear relationship with the logarithm of the CEA concentration (log*C*_CEA_) (*I* = −2.03 log*C*_CEA_ + 8.73, *R*^2^ = 0.993, inset in Figure 7a). The limit of detection (LOD), calculated based on a signal-to-noise ratio of 3 (S/N = 3), is 6.3 pg mL^−1^. A comparison between the determination of CEA using different methods is demonstrated in Table S2 (Supplementary Materials) [51,52,53,54,55,56,57]. The LOD was lower than that obtained using square wave voltammetry [51], chronoamperometric [52] or electrochemiluminescence [53] detection through sandwich immunoassay, or chemiluminescence determination based on aptamer functionalized magnetic silicon composite (Apt/AuNPs/PDDA-SiO_2_@Fe_3_O_4_) [54], or EIS determination based on the immobilization of the Ab on the poly(ethyleneglycol)-NH_2_-connected, pyrenebutyric acid-functionalized, graphene/gold nanoparticle-modified GCE (BSA/Ab/AuNPs/PPYGR/GCE) [55], or an 11-mercaptoundecanoic-acid-grafted chitosan and poly(N-methylaniline)-modified screen-printed carbon electrode (BSA/Ab/tCHI/dPNMA/SPCE) [56], but higher than that of an aptasensor based on Fe-MOF-supported self-polymerized dopamine-decorated Au NPs (NH_2_-aptamer/Au@PDA@Fe-MOF/GCE) [57].

### 2.7. Selectivity, Reproducibility, and Stability of the Immunosensor

To validate the selectivity of the immunosensor, the effect of common tumor biomarkers, including prostate specific antigen (PSA), carbohydrate antigen 19-9 (CA19-9), alpha-fetoprotein (AFP), carbohydrate antigen 12-5 (CA12-5), and neutrophil gelatinase-associated lipocalin (NGAL), was investigated as potential interferents. As shown in Figure 7b, when these substances at 10 times their concentrations were incubated with the immunoelectrode, the DPV signal of the probe remained almost unaffected. However, in the presence of CEA or a mixture containing CEA, the signal of the redox probe significantly decreased. These results demonstrate the selectivity of the immunosensor, attributed to the specific recognition by the biological antibodies. In addition, seven immunosensors were prepared simultaneously and incubated with CEA (1 ng mL^−1^). The DPV signals of the obtained electrodes were examined in Fe(CN)_6_^3-/4-^ solution. As shown in Figure 7c, the relative standard deviation (RSD) was 1.8%, demonstrating high reproducibility in the electrode preparation process. The storage stability of the immunosensor with or without the formed immunoassay was also investigated. On the one hand, the immunosensor was incubated with CEA (1 ng mL^−1^) and the resulting electrode was stored at 4 °C for different numbers of days. Then, the DPV curve of the electrode was measured in Fe(CN)_6_^3-/4-^ solution. As shown in Figure 7d, the signal response of the electrode remained at 99.9%, 97.8%, 96.0%, 94.0%, and 91.4% of the initial response after 1 to 5 days of storage, confirming the good storage stability of both the immobilized antibody and the formed immunocomplex. After storage for 6 days, the signal remained at 88.2%. On the other hand, the immunosensors without CEA binding were stored at 4 °C for 10 days. The signal for CEA (1 ng mL^−1^) detection was 94.1% of that obtained using the freshly prepared immunosensor, indicating the high stability of the immobilized antibody. 

### 2.8. Real Sample Analysis

To assess the potential application of the developed immunosensor, fetal bovine serum was selected as the real sample, and a standard addition method was employed to determine the concentration of the CEA. Specifically, different concentrations of CEA were added to simulate samples with varying CEA concentrations. As shown in Table 1, the recovery rates of the immunosensor ranged from 96.3% to 101%, with a low relative standard deviation (less than 2.9%), indicating that the immunosensor exhibits excellent reliability and accuracy in practical applications.

## 3. Materials and Methods

### 3.1. Chemicals and Materials

Carcinoembryonic antigen (CEA), anti-CEA antibodies, alpha-fetoprotein (AFP), carbohydrate antigen 125 (CA 125), neutrophil gelatinase-associated lipocalin (NGAL), carbohydrate antigen 199 (CA 19-9), and fetal bovine serum were purchased from Beijing KeyGen Biotech Co., Ltd. (Beijing, China). S100 calcium-binding protein (S100) was obtained from Wuhan Sanying Biotechnology Co., Ltd. (Wuhan, China). Prostate specific antigen (PSA) was procured from Beijing Biodragon Immunotechnologies Co., Ltd. (Beijing, China). Tetraethyl orthosilicate (TEOS, 98%), 3-aminopropyltriethoxysilane (APTMS), cetyltrimethylammonium bromide (CTAB), bovine serum albumin (BSA), glutaraldehyde (GA), sodium dihydrogen phosphate dihydrate (NaH_2_PO_4_·2H_2_O), disodium hydrogen phosphate dodecahydrate (Na_2_HPO_4_·12H_2_O), sodium hydroxide (NaOH), potassium ferricyanide (K_3_Fe(CN)_6_), and potassium ferrocyanide (K_4_Fe(CN)_6_) were purchased from Aladdin Bio-Chem Technology Co., Ltd. (Shanghai, China). Sulfuric acid, acetone, anhydrous ethanol (99.8%), and concentrated hydrochloric acid (HCl, 36-38%) were obtained from Shuanglin Reagent Co., Ltd. (Hangzhou, China). Phosphate-buffered saline (PBS, 0.01 M, pH = 7.4) was prepared using sodium dihydrogen phosphate and disodium hydrogen phosphate. All chemical reagents were of analytical grade.

### 3.2. Characterization and Instrumentation

The morphology and thickness of NH_2_-VMSF were characterized using transmission electron microscopy (TEM, model HT7700, Hitachi, Tokyo, Japan). To prepare TEM samples, the VMSF layer was carefully scraped off the electrode using a scalpel and dispersed in anhydrous ethanol with subsequent ultrasonic dispersion. Subsequently, the resulting dispersion was drop-cast onto a copper grid. Before morphology characterization under 200 kV, the sample was allowed to air dry naturally. All electrochemical experiments, including cyclic voltammetry (CV), electrochemical impedance spectroscopy (EIS), and differential pulse voltammetry (DPV), were conducted on an Autolab electrochemical workstation (model PGSTAT302N, Metrohm Autolab, Utrecht, Switzerland). A conventional three-electrode system was employed, with Ag/AgCl as the reference electrode, platinum wire as the counter electrode, and the modified electrode as the working electrode. The frequency range for EIS measurements was from 0.1 Hz to 100 kHz, with a perturbation amplitude of 5 mV. X-ray photoelectron spectroscopy (XPS) measurements were performed under 250 W, 14 kV, with Mg Kα radiation (PHI5300, Physical Electronics, Chanhassen, MN, USA).

### 3.3. Preparation of NH_2_-VMSF/p-GCE Electrode

Before use, GCE (with a diameter of 3 mm) was polished using 0.5 μm, 0.3 μm, and 0.05 μm aluminum oxide powders successively. Subsequently, the electrode was ultrasonically cleaned with ethanol and ultrapure water, respectively. The cleaned GCE was then pre-activated using electrochemical polarization. Specifically, a constant potential of +1.8 V was applied to GCE for 300 s for anodic oxidation. Then, cyclic voltammetry scans were performed in PBS (0.1 M, pH = 5) from −1.3 V to +1.25 V for cathodic polarization. The resulting electrode is referred to as p-GCE.

Then, NH_2_-VMSF was grown on the surface of p-GCE electrode using the electrochemically assisted self-assembly method (EASA) method as reported in the literature [58,59]. Briefly, a mixture of 20 mL ethanol, 20 mL sodium nitrate solution (0.1 M, pH = 2.36), CTAB (1.585 g), and APTES (318 μL) was prepared. Then, the pH of the solution was adjusted to 2.97 using HCl before TEOS (2732 μL) was added. Then, the mixture was vigorously stirred and reacted for 2.5 h to obtain the precursor solution. Subsequently, the p-GCE electrode was used as the working electrode and immersed in the precursor solution. Then, a constant current (current density: −0.74 mA/cm^2^, duration: 10 s) was applied to achieve the rapid growth of NH_2_-VMSF. Afterwards, the electrode was rapidly rinsed with ultrapure water. After drying with nitrogen gas, the electrode was aged overnight at 80 °C, resulting in an electrode with surfactant micelles (SMs) inside the nanochannels, denoted as (SM@NH_2_-VMSF/p-GCE). Finally, SM@NH_2_-VMSF/p-GCE electrode was immersed in an HCl–ethanol solution (0.1 M) and stirred for 5 min to facilitate the removal of micelles, yielding an electrode with open channels, referred to as NH_2_-VMSF/p-GCE electrode.

### 3.4. Fabrication of the Immunosensor

To prepare the immunosensor, glutaraldehyde (GA) was used as a cross-linking agent to derivatize the amino groups on the outer surface of NH_2_-VMSF to aldehyde groups for covalent immobilization of antibodies (Ab). To ensure that GA was selectively modified on the outer surface of NH_2_-VMSF, amino groups on the outer surface were derivatized with aldehyde before removing SM. Specifically, SM@NH_2_-VMSF/p-GCE electrode was immersed in a GA solution (1%, 10 μL) and incubated in the dark at 37 °C for 10 min. After the electrode was thoroughly rinsed, it was soaked in an HCl–ethanol solution (0.1 M) and stirred for 5 min to remove micelles. The obtained electrode was designated as GA/NH_2_-VMSF/p-GCE electrode. Then, the aldehyde-functionalized electrode was immersed in CEA antibody solution (100 μg/mL in 0.01 M PBS, pH = 7.4) and incubated at 4 °C for 1 h [60]. Afterward, the electrode was thoroughly washed with PBS (0.01 M, pH = 7.4) to remove unbound antibodies from the electrode surface. Subsequently, BSA (0.1 wt%) was used to block non-specific sites by incubating at 4 °C for 30 min. After thorough washing, the resulting immunosensing electrode was denoted as BSA/Ab/GA/NH_2_-VMSF/p-GCE.

### 3.5. Electrochemical Detection of CEA

The medium for binding the immunorecognition interface with CEA was PBS (0.01 M, pH = 7.4). The immunosensors were incubated with different concentrations of CEA at 4 °C for 60 min, respectively. The detection solution was potassium chloride (0.1 M) with Fe(CN)_6_^3-/4-^ (2.5 mM). DPV was employed to measure the electrochemical signal of Fe(CN)_6_^3-/4-^ in the electrolyte before and after CEA binding. For the determination of CEA in serum, serum samples from healthy individuals were diluted 50 times with PBS (0.01 M, pH = 7) before undergoing electrochemical detection.

## 4. Conclusions

In this work, an electrochemical immunosensor was successfully developed for the rapid and highly sensitive electrochemical detection of the tumor marker carcinoembryonic antigen (CEA). A pre-activated glassy carbon electrode (p-GCE) was obtained using a simple electrochemical polarization method, eliminating the need for the introduction of an adhesion layer for stable modification with amino-modified VMSF (NH_2_-VMSF) on the p-GCE surface. When the nanochannels were filled with micelles, the amino groups on the outer surface of the NH_2_-VMSF were functionalized with aldehyde, enabling the covalent immobilization of antibodies. The NH_2_-VMSF facilitated the migration of negatively charged redox probes to the supporting electrode. In the presence of the target CEA, the formation of an immunocomplex might hinder the migration of the redox probe and increased the interfacial resistance, leading to the phenomenon of immunorecognition-induced signal decrease. Based on this principle, the sensitive electrochemical detection of CEA was achieved. The developed immunosensor possesses the advantages of simplicity in preparation, high sensitivity in detection, and excellent selectivity, demonstrating great potential for the sensitive electrochemical detection of tumor biomarkers.

## Figures and Tables

**Table 1:** Detection of CEA in fetal bovine serum sample using standard addition method.

Sample	Spiked ^b^ (ng mL^−1^)	Found (ng mL^−1^)	RSD (%, n = 3)	Recovery (%)
Serum ^a^	0.0100	0.00963	1.3	96.3
	0.100	0.0978	2.8	97.8
	1.00	1.01	2.9	101

^a^ Samples with added CEA were diluted by a factor of 50 using the electrolyte. ^b^ The concentration of CEA was the concentration after dilution.

## Data Availability

The data presented in this study are available on request from the corresponding author.

## Supplementary Materials

The supporting information can be downloaded at: https://www.mdpi.com/article/10.3390/molecules29040858/s1.
