# Supplementary material for: Sensitive Electrochemical Detection of Carcinoembryonic Antigen Based on Biofunctionalized Nanochannel Modified Carbonaceous Electrode
Source: Molecules. 2024 Feb 15;29(4):858. doi: 10.3390/molecules29040858 (PMC10892148; doi:10.3390/molecules29040858)
Supplement: Supplementary file 1 [file molecules-29-00858-s001.zip › molecules-2784161-supplementary.pdf]

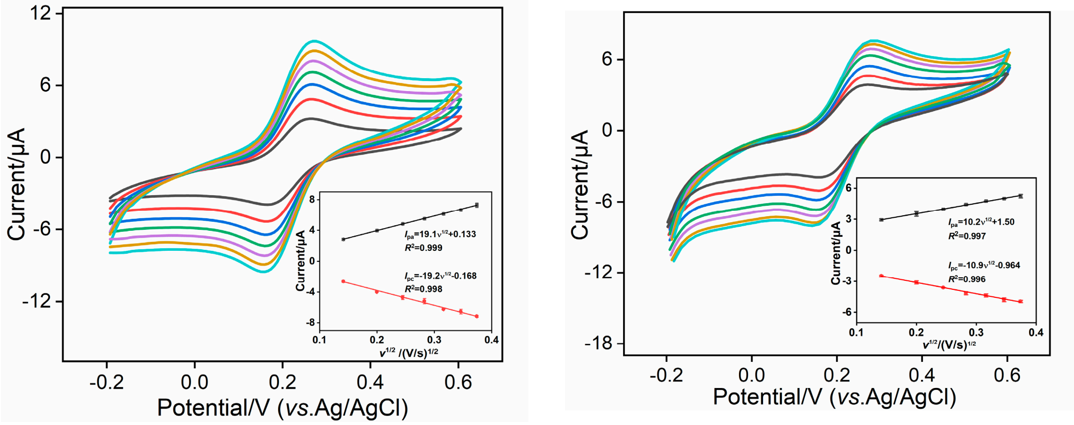

(a) (b)

Figure S1 CV curves obtained on p-GCE (a) and bare GCE (b) at different scan rate (20, 40, 60, 80, 100, 120, 140 mV/s, from) with 0.5 mM $K_3[Fe(CN)_6]$ containing 0.1 M KCl. The inset is the linear regression curve between peak current vs square root of scan rate derived from the CV curves.

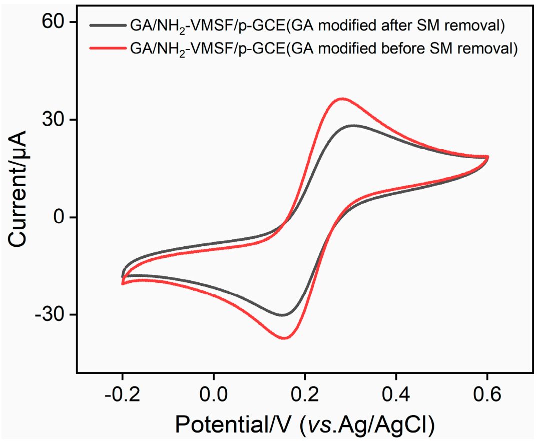

Figure S2. CV curves obtained electrodes obtained using GA modification before or after SM removal in 0.1 M KCl containing 2.5 mM $Fe(CN)_6^{3/4-}$.

Table S1. Ratios of C-O, C=O, O-C=O peak area relative to C-C/C=C peak area in different electrode.

electrode	ratios of C-O, C=O, O-C=O peak area relative to C-C/C=C peak area (%)		
	C-O	C=O	O-C=O
GCE	10.5	5.08	6.60
Electrode after anodic polarization	38.6	6.68	5.91
p-GCE	24.6	6.48	8.43

Table S2. Comparison between CEA detection performance using different method.

Materials	***Method***	***Liner range (ng/mL)***	***LOD (pg/mL)***	*Ref.*
AuNPs-pMCP-Ab$_2$/CEA/BSA/ Ab$_1$/Au/3D-G/GCE	SWV	0.5-200	310	[51]
HPR-Ab/CEA/BSA/lectin/cysteamine/AuNPs /SPCE	CA	0.5-10	10	[52]
GCE/S-GO/SA-AuNPs/Ab$_1$/ CEA/Ab$_2$-SA-SNPs-Lu	ECL	0.1-5	58	[53]
Apt/AuNPs/PDDA-SiO$_2$@Fe$_3$O$_4$	CL	0.1−500	32	[54]
BSA/Ab/AuNPs/PPYGR/GCE	EIS	0.1-10^3	60	[55]
BSA/Ab/tCHI/dPNMA/ SPCE	DPV	0.01-30	10	[56]
BSA/NH$_2$-aptamer/ AuNPs@PDA@ Fe-MOF/GCE	LSV	10^{-6}-10^3	3.3×10^{-4}	[57]
BSA/Ab/GA/NH$_2$-VMSF/ p-GCE	DPV	0.01-100	6.3	This work

AuNPs, gold nanoparticles; pMCP, poly-m-Cresol purple; Ab$_2$, the secondary anti-CEA; BSA, bovine serum albumin; Ab$_2$, the first anti-CEA; 3D-G, three dimensional graphene; GCE, glassy carbon electrode; SWV, square wave voltammetry; HPR-Ab, horseradish peroxidase-labeled anti-CEA; GA, Chronoamperometric; SPCE, screen-printed carbon electrodes; PDA, self-polymerized dopamine; MOF, metal-organic frameworks; LSV, linear sweep voltammetry; S-GO, thiolated graphene oxide; SA-AuNPs, streptavidin-coated gold nanoparticles; SA-SNPs-Lu, silver NPs coated with luminol and streptavidin; ECL, electrochemiluminescence; Apt, aptamer; PDDA, polydiallyl dimethylammonium chloride; CL, chemiluminescence; PPYGR, poly(ethyleneglycol)-NH$_2$/pyrenebutyric acid functionalized graphene; EIS, electrochemical impedance spectroscopy; tCHI, 11-mercaptoundecanoic acid grafted chitosan; dPNMA, doped poly(N-methylaniline).
